# Wenshen Yiqi Keli Mitigates the Proliferation and Migration of Cigarette Smoke Extract-Induced Human Airway Smooth Muscle Cells through miR-155/FoxO3a Axis

**DOI:** 10.1155/2022/4427637

**Published:** 2022-11-22

**Authors:** Lei Dong, Hongli Li, Minjing Li, Liting Zhang, Chenhui Qiu, Ye Chen

**Affiliations:** ^1^Department of Respiratory and Critical Care Medicine, The Second Affiliated Hospital of Zhejiang Chinese Medical University, Hangzhou, Zhejiang, China; ^2^The Second Clinical Medicine College of Zhejiang Chinese Medical University, Hangzhou, Zhejiang, China

## Abstract

Some domestic scholars revealed the effectiveness of Wenshen Yiqi Keli (WSYQKL) on chronic obstructive pulmonary disease (COPD). However, the exact mechanism of WSYQKL on COPD is fuzzy and needs further research. We adopted UPLC-Q/TOF-MS to analyze the chemical components of WSYQKL. In *in vitro* experiments, human airway smooth muscle cells (hASMCs) were intervened with 2.5% cigarette smoke extract (CSE), medicine serum of WSYQKL, miR-155 mimic, and FoxO3a silencing. Cell viability, proliferation, migration, and the expressions of miR-155, PCNA, Ki67, p21, p27, and FoxO3a were examined by cell counting kit-8, EdU staining, Transwell assay, scarification assay, qRT-PCR, immunol cytochemistry, and western blot, respectively. The association between miR-155 and FoxO3a was assessed by database and luciferase reporter gene analysis. We identified 47 kinds of chemical compositions of WSYQKL in ESI^+^ mode and 42 kinds of components of WSYQKL in ESI^−^ mode. The medicine serum of WSYQKL strongly alleviated the proliferation and migration of hASMCs induced by CSE in a concentration-dependent manner. The medicine serum of WSYQKL enhanced the levels of p21, p27, and FoxO3a and weakened PCNA and Ki67 levels in hASMCs induced by CSE with the increase of concentration. MiR-155 mimic or FoxO3a silencing notably advanced CSE-treated HASMC viability, proliferation, migration, and the levels of PCNA and Ki67 and downregulated the levels of p21, p27, and FoxO3a in CSE-triggered hASMCs, which was reversed by WSYQKL-containing serum. Our results described that WSYQKL alleviated the proliferation and migration of hASMCs induced by CSE by modulating the miR-155/FoxO3a axis.

## 1. Introduction

Chronic obstructive pulmonary disease (COPD) is a familiar preventable and treatable disease marked by persistent respiratory symptoms and airflow restriction [1]. Respiratory symptoms and airflow restrictions are caused by airway and/or alveolar abnormalities caused by toxic particles or gases [2]. Airflow restriction is the major reason for clinical symptoms in COPD patients, and the cardinal pathological feature of airflow restriction is airway remodeling [3, 4]. Airway remodeling is the primary pathological basis of COPD and a pivotal factor in the sustainable development of COPD [5]. The process of airway remodeling involves multiple cells and cytokines, among which airway smooth muscle cells (ASMCs) are particularly considerable and the most in-depth research [6]. Excessive proliferation of ASMCs causes tracheal dysfunction and is participated in airway remodeling and airway hyperresponsiveness [7]. To date, there are no effective COPD treatments for inhibiting cell proliferation of ASMCs and no globally accepted criteria for defining early COPD [8, 9]. Therefore, there is an urgent need for new biomarkers for the early detection of COPD to facilitate the timely intervention of the disease.

MicroRNA (miRNA) is an endogenous noncoding RNA that can cleave mRNA to inactivate it and weaken the translation process [10]. Regulation of cellular activities such as proliferation, apoptosis, and differentiation by altering the expression of miRNAs has been implicated in the development of various diseases including cardiovascular dysfunction, lung disease, and cancer [11,12]. It has been reported that miRNA can modulate the pathological process of COPD [13,14]. Among them, miR-155 was reported to be overexpressed in bronchial epithelial cells mediated by PM2.5 [15]. However, the exact mechanism of miR-155 in COPD is fuzzy.

Now, mounting clinicians realize that traditional Chinese medicine (TCM) has its unique advantages in the treatment of COPD. Wenshen Yiqi Keli (WSYQKL) is a commonly used prescription for the clinical treatment of COPD by Professor Xu Zhiying of TCM. WSYQKL is composed of *Astragali Radix*, *Cuscutae Semen*, *Fluoritum*, *Paris polyphylla*, and *Rhizoma Curcumae*. The long-term clinical practice indicated that WSYQKL can ameliorate and maintain the pulmonary function of COPD patients, mitigate clinical symptoms and decrease the number of acute attacks, with good social and economic benefits [16,17]. However, whether WSYQKL affects the proliferation and migration of cigarette smoke extract-induced human airway smooth muscle cells by modulating miR-155 expression has not been reported.

In this study, we used human ASMCs (hASMCs) as target cells to investigate whether WSYQKL-containing serum affects the proliferation and migration of cigarette smoke extract (CSE)-triggered hASMCs by regulating miR-155, and hoped to clarify its specific regulatory mechanism and provide new ideas for the diagnosis and treatment of COPD.

## 2. Materials and Methods

### 2.1. Preparation of WSYQKL Test Solution

We took an appropriate amount of granules and grind them to powder, and then precisely weighed 1.00 g of the powder. After adding 25 mL of methanol, the above powder was extracted by ultrasonic for 45 min. After centrifugation (14000 r/min, 20 min), we obtained the supernatant, namely, the test solution.

### 2.2. UPLC-Q-TOF-MS Analysis of WSYQKL

The WSYQKL test solution was analyzed on a UHPLC (Waters Corporation, USA) equipped with a C18 column (ACQUITY UPLC® BEH C18, 150 × 2.1 mm, 1.7 *μ*m). The mobile phase was with 0.1% formic acid acetonitrile (*A*) and 0.1% formic acid water (*B*) in a program of elution: 99%∼80% B at 0–4 min; 80%–45% B at 4–16 min; 45%–35% B at 16–18 min; 35%–1% B at 18–20 min; 1% B at 20–22 min. The flow rate, injection tray temperature, column temperature, and injection volume were set to 0.3 mL/min, 8°C, 40°C, and 2 *μ*L, respectively.

The time-of-flight MS was done in ESI^+^ and ESI^−^ mode using TurboIonSpray ion source. Gas1: 55; Gas2: 55; CUR: 35; source temperature: 600°C; ISVF: 5500 V/-4500 V (ESI^+^ and ESI^−^ mode); TOF-MS scan m/*z* range: 50–1500 Da; production scan m/*z* range: 25–1000 Da; TOF-MS scan accumulation time: 0.25 s/spectra; product ion scan accumulation time: 0.035 s/spectra. Secondary MS was acquired by IDA and high sensitivity mode; DP: ± 60 V (ESI^+^ and ESI^−^ mode); collision energy: 35 ± 15 eV; IDA settings were as follows: exclude isotopes within 4 Da; candidate ions to monitor per cycle 12.

### 2.3. Animal Care

Twenty male SD rats of specific pathogen-free (SPF) grade, weighing 200 ± 20 g, were purchased from Shanghai SLAC Laboratory Animal Co., Ltd. The Animal Ethics Committee of Zhejiang Eyong Pharmaceutical Research and Development Center granted the animal experiments. They were adapted for 7 days in a breeding room (20 ± 2°C, 55 ± 5%, and 12-h light/dark cycle).

### 2.4. Preparation of WSYQKL-Containing Serum

WSYQKL was bought from Zhejiang Hospital of TCM. Specifications: 15 g/pack, 1 pack each time, 2 times a day. A total of 20 rats were randomly distributed into the control group and WSYQKL group, 10 per group. Rats in the WSYQKL group were administered by gavage at a dose of 3.2 g/kg/d, twice a day, for 5 consecutive days. The drug doses for rats were determined using the following formula: Rats (g/kg) = [human dose (30 g/day)/human weight (60 kg)] × 6.3. The rats in the control group were given an equal volume of normal saline. Two hours after the last administration, the blood samples of the rats were obtained by eye enucleation. After standing for 1 h, the blood samples were centrifuged and filtered followed by inactivation in a 56°C water bath for 30 min and then stored at −80°C.

### 2.5. Collection of Cigarette Smoke Extract (CSE)

We ignited a cigarette (11 mg tar, 0.9 mg nicotine, and 11 mg carbon monoxide). One end of a rubber tube was connected to a cigarette filter, and the other end was connected to a syringe, to mimic the actual smoking situation of a person. 300 mL of smoke from the syringe was injected into a 10 mL glass bottle of DMEM, which was shaken to dissolve into the culture medium. We obtained 100% stock solution CSE (30 mL of smoke per mL of DMEM).

### 2.6. Cell Culture

hASMCs (C1158) were bought from WHELAB (China). For the culture of hASMCs, MEM medium (M0300, WHELAB, China) supplied with 10% FBS was used. hASMCs were put in a cell incubator (Herocell 180, RADOBIO, China).

### 2.7. Cell Transfection

MiR-155 mimic (miR10004658-1-5), miR-155 negative control (miR-155_NC, miR1N0000002-1-5), miR-155 inhibitor (miR20004658-1-5), and miR-155 inhibitor_NC (miR2N0000002-1-5) were bought from RiboBio (China). Small interfering RNA FoxO3a (si-FoxO3a, abx917150) and si-FoxO3a_NC (abx941273) were bought from Abbexa (USA). For the transfection of hASMCs, Lipo6000 (C0526, Beyotime, China) was utilized.

### 2.8. Study Design

To analyze the role of WSYQKL in hASMCs induced by 2.5% CSE, hASMCs were allocated to the control group (20% normal rat serum (NRS) culture), 2.5% CSE group (cells were intervened with 20% NRS for 24 h prior to 2.5% CSE solution treatment for 48 h), 2.5% CSE + 5% WSYQKL-containing serum group (cells were stimulated with 5% WSYQKL-containing serum and 15% NRS for 24 h before exposing to 2.5% CSE solution), 2.5% CSE + 10% WSYQKL-containing serum group (cells were treated with 10% WSYQKL-containing and 10% NRS for 24 h before exposing to 2.5% CSE solution), and 2.5% CSE + 20% WSYQKL-containing serum group (cells were intervened with 20% WSYQKL-containing serum and then subjected to 2.5% CSE solution). Then, to check whether miR-155/FoxO3a axis affects the regulation of WSYQKL on 2.5% CSE-mediated hASMCs, hASMCs were allocated to the 2.5% CSE + miR-155_NC group, 2.5% CSE + miR-155 mimic group, 2.5% CSE + miR-155 mimic +20% WSYQKL-containing serum group, 2.5% CSE + si-FoxO3a_NC group, 2.5% CSE + si-FoxO3a group, and 2.5% CSE + si-FoxO3a +20% WSYQKL-containing serum group.

### 2.9. Cell Viability Analysis of HASMCs

For the examination of HASMC cell viability, CCK-8 Kit (HY-K0301, MCE, USA) was earmarked. HASMCs (1 × 10^4^/well) were loaded in ninety-six-well plates. The next day, cells were processed based on the above study design for 24 h. After that, we removed the cell medium and then dripped 10 *μ*L CCK-8 solution into each well. After being reacted at 37°C for 4 h, we recorded the absorbance (450 nm) utilizing a microplate reader (CMaxPlus, MD, USA).

### 2.10. Cell Proliferation Analysis of HASMCs

For the proliferation of hASMCs, we utilized Edu-594 kit C0078S, Beyotime, China). hASMCs (1 × 10^4^/well) were appended to twelve-pore plates containing coverslips. After adhesion, cells were intervened based on the above study design for 48 h. Thereafter, 2 × EdU solution was injected into each well (37°C, 4 h). Then, 95% ethanol was employed to fix cells (37°C, 15 min). After rinsing, permeabilization buffer (P0097, Beyotime, China) was added (37°C, 10 min). After rinsing again, the Click solution was added (37°C, 0.5 h, darkroom). DAPI was exploited to identify the nucleus (37°C, 10 min, dark room). For the observation of the proliferative ability, a fluorescence microscope was used.

### 2.11. Migration Analysis of HASMCs

hASMCs were serum-starved for 24 h and then subjected to digestion and centrifugation. Cell precipitation was resuspended in a medium without FBS and appended to the apical chamber (3422, Corning, USA). The media with 10% FBS was dripped into the basolateral chamber. After 48 h of conventional culture, we removed hASMCs that did not migrate through the inner surface of the apical chamber. The fixation of hASMCs migrated into the lower compartment was done with methanol. Then, 0.1% crystal violet (C0775) offered by Sigma-Aldrich (USA) was applied to stain hASMCs. An optical microscope (IX71, Olympus, Japan) was employed to observe the number of migrated hASMCs. For scarification assay, a total of 8000 hASMCs were loaded in six-pore plates. The next day, the layer of hASMCs was injured with a 10 *μ*L pipette tip. We utilized PBS to rinse the floating hASMCs. Thereafter, MEM medium without serum was added to each well. The scratched results were taken at 0 h and 48 h and observed by the optical microscope. The relative migration distance was assessed utilizing Image J (version 1.48).

### 2.12. Immunol Cytochemistry Analysis

hASMCs loaded in 12-pore plates were intervened according to the above study design. Subsequently, fixation was conducted with 4% paraformaldehyde (P1110, Solarbio, China). Thereafter, a blocking Buffer (P0102, Beyotime, China) was applied to seal hASMCs (4°C, all night). Then, the anti-Ki67 antibody (1 : 1000, ab92742, Abcam, UK) and anti-PNCA antibody (1 : 250, ab92552, Abcam, UK) were added (4°C, all night). Then, anti-rabbit IgG H&L (HRP, 1 : 200, ab97051, Abcam, UK) was added (37°C, 60 min). After that, the hASMCs of each well were monitored under the optical microscope.

### 2.13. Western Blot Analysis

To extract proteins from hASMCs, we used RIPA buffer (abs9229, absin, China). After quantification of the extracted proteins, denaturation was performed. The protein was harvested for electrophoresis. After being electroblotted onto a nitrocellulose membrane, 5% BSA was utilized to seal the immunoblot (37°C, 120 min). Afterward, primary antibodies were added (4°C, 12 h). Then, the membrane was immersed in anti-rabbit IgG H&L (1 : 1000) at 37°C for another 1 h. A color reagent (1705061, BIO-RAD, USA) was then added. The blots were observed under an Imaging System (Odyssey CLx, LI-COR, USA). The primary antibodies of Ki67 (1 : 5000), PCNA (1 : 10000), p21 (1 : 10000, ab109520), p27 (1 : 5000, ab32034), FoxO3a (1 : 10000, ab109629), and GAPDH (1 : 10000, ab181602) were bought from Abcam (UK).

### 2.14. Real-Time Quantitative PCR

For the isolation of RNA from hASMCs, the miRNA extraction isolation kit (DP501) provided by TIANGEN (China) was utilized. To synthesize cDNA, we used miRNA cDNA first-strand synthesis kit (KR211) bought from TIANGEN (China). Then, qRT-PCR was carried out utilizing a miRNA qPCR kit (FP411, TIANGEN, China) in a PCR system (CFX96, Bio-Rad, USA). Gene-specific primers were as follows: miR-155 forward: 5′‐GGCTAAGGAGATTGGTGCTGTA-3′, Reverse: 5′‐ACGAGGGGCTG-AGACATTTAC-3′; U6 forward: 5′‐AGTAAGCCCTTGCTGTCAGTG-3′, Reverse: 5′‐CCTGGGTCTGATAATGCTGGG-3′.

U6 was validated as the normalizer. The 2^−ΔΔCt^ was employed for data evaluation.

### 2.15. Luciferase Reporter Gene Analysis

We forecasted the binding sites between FoxO3a and miR-155. MiR-155 mimic or miR-155-NC was co-transfected into hASMCs with a pmirGLO vector (E1330) offered by Promega (USA) containing WT or MUT 3′-UTR of FoxO3a utilizing Lipo6000. After 48 h, to assess the luminescence signal, we used Dual-Lucy Assay Kit (D0010, Solarbio, China).

### 2.16. Statistical Analysis

All analyses were computed utilizing SPSS 16.0 software. The differences among multiple groups were compared via one-way ANOVA followed by an SNK test when data satisfied a normal distribution. Kruskal–Wallis H test was performed for those with uneven variance. Data were indicated as mean ± SD. Statistically, *P* < 0.05 was meaningful.

## 3. Results

### 3.1. The Base Peak Chromatograms of WSYQKL

**Figure 1:**
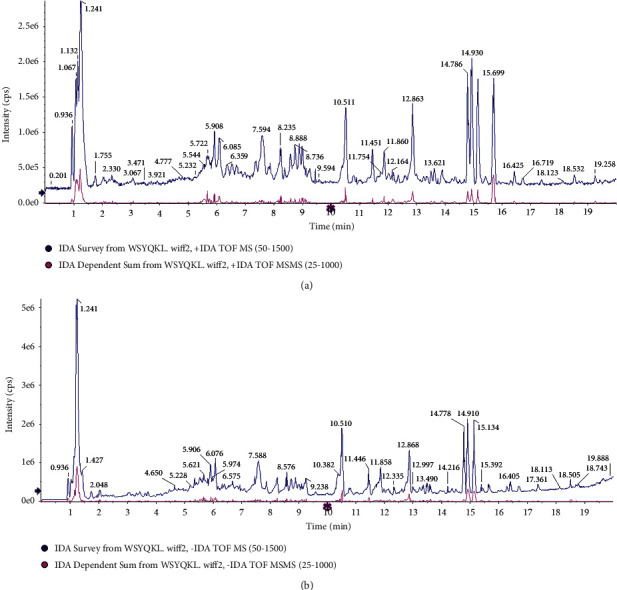
The base peak chromatograms of chemical components of WSYQKL in UPLC-Q/TOF-MS analysis. (a) Base peak ion chromatogram of WSYQKL in positive mode. (b) Base peak ion chromatogram of WSYQKL in negative mode.

UPLC-Q/TOF-MS was employed to qualitatively identify the chemical components of WSYQKL. The total ion chromatogram of WSYQKL was displayed in Figure 1(a) (positive ion mode) and Figure 1(b) (negative ion mode).  Table S1 presented 47 kinds of the chemical composition information of WSYQKL in ESI^+^ mode.  Table S2 presented 42 kinds of components of WSYQKL in ESI^−^ mode.

### 3.2. WSYQKL-Containing Serum Inhibited Cell Proliferation on CSE-Mediated HASMCs

**Figure 2:**
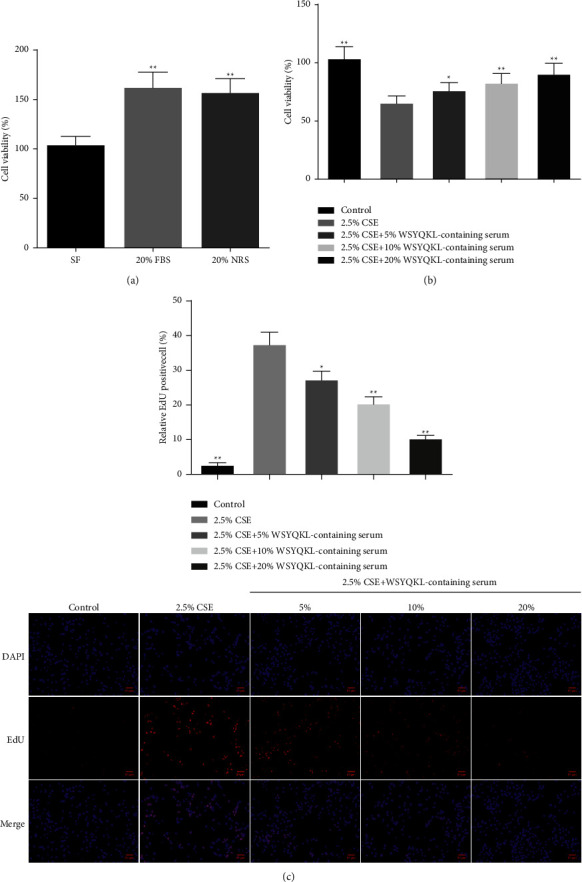
Effects of the WSYQKL-containing serum on cell proliferation in CSE-mediated HASMCs. (a) Cell counting kit-8 was utilized to measure the cell viability of HASMCs treated with Dulbecco's Modified Eagle's Medium supplemented with serum-free (SF), 20%FBS, and 20% normal rat serum (NRS). (b) Cell viability of CSE-mediated HASMCs with 20% WSYQKL-containing serum treatment was measured using a CCK-8 assay. (c) Cell proliferation of CSE-mediated HASMCs with 20% WSYQKL-containing serum treatment was assessed by EdU assay. ^*∗*^*P* < 0.05, ^*∗∗*^*P* < 0.01*vs* SF or 2.5% CSE group.

We first treated hASMCs with 20% FBS and 20% NRS. As displayed in Figure 2(a), hASMC**s** viability in the 20% FBS group and 20% NRS group was higher relative to the serum-free group, and there was no difference between the two groups. Therefore, 20% NRS can be used instead of 20% FBS (*P* < 0.01). Next, by performing CCK-8 and EdU staining experiments, 2.5% CSE induced the decrease of hASMCs viability and promoted the EdU-positive cell levels, while the medicine serum of WSYQKL effectively reversed its effect in a concentration-dependent manner (Figures 2(b) and 2(c), *P* < 0.05).

### 3.3. WSYQKL-Containing Serum Inhibited Migration on CSE-Triggered HASMCs

**Figure 3:**
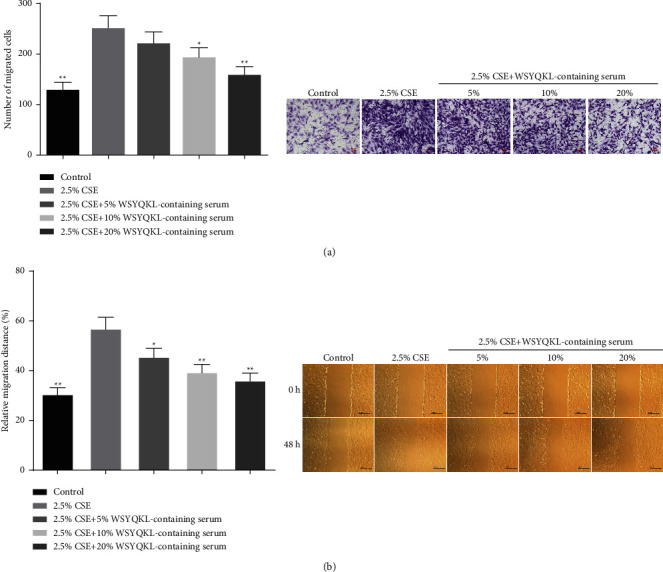
Effects of the WSYQKL-containing serum on cell migration in CSE-mediated HASMCs. Cell migration of CSE-mediated HASMCs with 20% WSYQKL-containing serum treatment was assessed using the (a) Transwell and (b) wound healing assays. ^*∗*^*P* < 0.05, ^*∗∗*^*P* < 0.01*vs* 2.5% CSE group.

To check the role of WSYQKL on the migration of hASMCs induced by CSE, we conducted a Transwell assay and scarification assay. The results demonstrated that 2.5% CSE obviously facilitated the migration of hASMCs (Figures 3(a) and 3(b), *P* < 0.01). More importantly, we discovered that the medicine serum of WSYQKL strongly alleviated the migration of hASMCs induced by CSE in a concentration-dependent manner (Figures 3(a)and 3(b), *P* < 0.01).

### 3.4. WSYQKL-Containing Serum Decreased Levels of Proliferation-Related Markers on CSE-Triggered hASMCs

**Figure 4:**
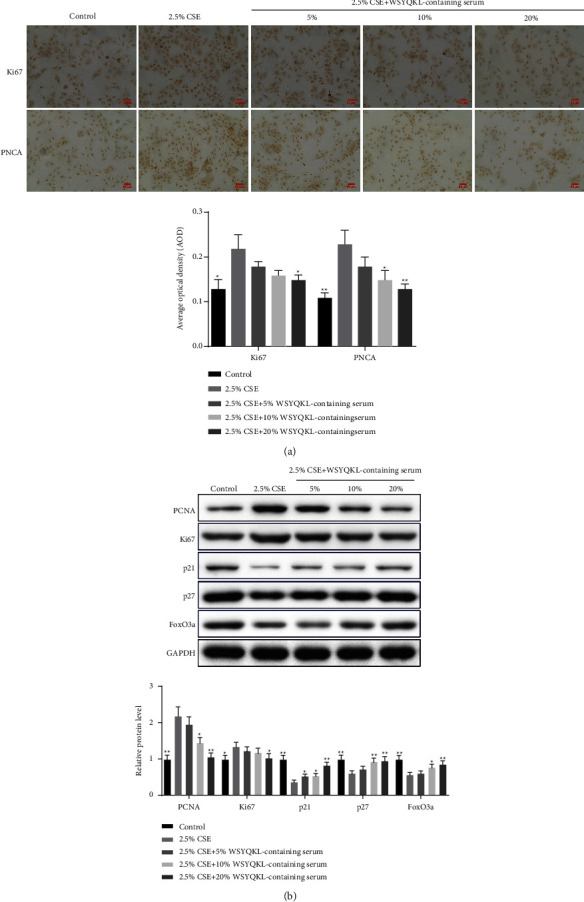
Effects of the WSYQKL-containing serum on cell proliferation-related protein levels in CSE-mediated hASMCs. (a) Immunohistochemical staining of Ki67 and PCNA in the CSE-mediated hASMCs treated with 20% WSYQKL-containing serum. Scale bar = 50 *μ*m. (b) The expression of the cell proliferation-related proteins, PCNA, Ki67, p21, and p27, as well as FoxO3a, was detected by western blotting after 20% WSYQKL-containing serum treatment. ^*∗*^*P* < 0.05, ^*∗∗*^*P* < 0.01*vs* 2.5% CSE group.

In this part, immunol cytochemistry was applied to examine the positive expressions of Ki67 and PCNA. We found an up-regulation of Ki67 and PCNA in hASMCs induced by CSE (Figure 4(a), *P* < 0.05). The medicine serum of WSYQKL down-regulated the expressions of Ki67 and PCNA in hASMCs induced by CSE with the increase of concentration (Figure 4(a), *P* < 0.05). The same results were found in the western blot assay (Figure 4(b), *P* < 0.05). Furthermore, the levels of p21, p27, and FoxO3a in the 2.5% CSE group were lower relative to the control group (Figure 4(b), *P* < 0.01). The medicine serum of WSYQKL enhanced the levels of p21, p27, and FoxO3a in HASMCs induced by CSE with the increase in concentration (Figure 4(b), *P* < 0.05).

### 3.5. WSYQKL-Containing Serum Reversed the Proproliferative Effect of miR-155 Mimic and si-FoxO3a on CSE-Mediated HASMCs

**Figure 5:**
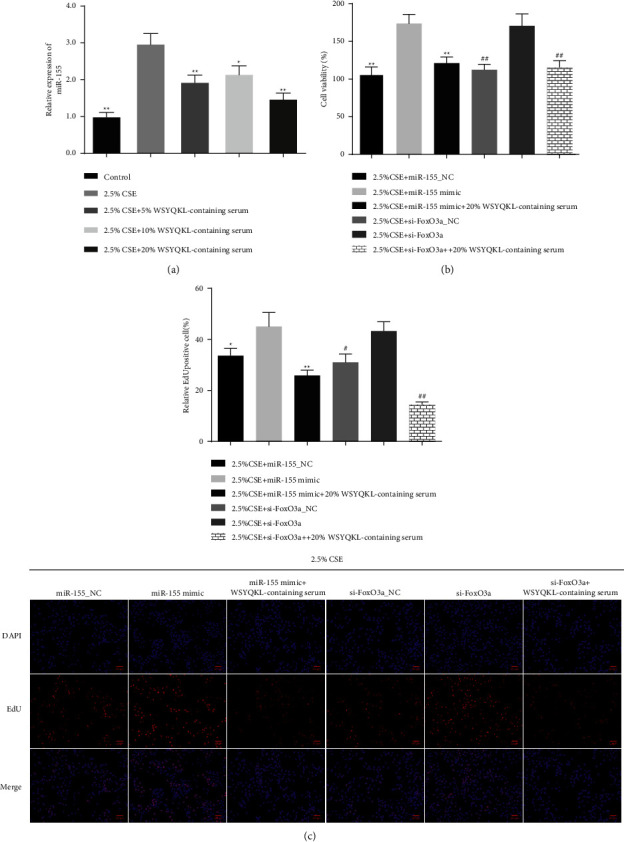
Effects of the WSYQKL-containing serum on cell proliferation in CSE-mediated hASMCs with miR-155 overexpression or FoxO3a knockdown. (a) The expression of miR-155 in the CSE-mediated hASMCs treated with 20% WSYQKL-containing serum was measured using a qRT-PCR assay. (b) Cell viability was confirmed after 20% WSYQKL-containing serum treatment in miR-155 mimic or si-FoxO3a transfected CSE-mediated hASMCs. (c) Edu assays were conducted to evaluate cell proliferative ability in miR-155 mimic or si-FoxO3a transfected CSE-mediated hASMCs. ^*∗*^*P* < 0.05, ^*∗∗*^*P* < 0.01*vs* 2.5% CSE + miR-155 mimic group. #*P* < 0.05, ##*P* < 0.01*vs* 2.5% CSE + si-FoxO3a group.

As presented in Figure 5(a), the qRT-PCR results described that a remarkable increase of miR-155 in CSE-treated hASMCs was found, while the medicine serum of WSYQKL counteracted its effect (*P* < 0.05). To probe whether WSYQKL affects the effect of miR-155 mimic or si-FoxO3a on CSE-mediated hASMCs, we conducted a series of cell experiments. MiR-155 mimic or si-FoxO3a notably advanced CSE-treated HASMC viability and proliferation, whereas the medicine serum of WSYQKL partially offset miR-155 mimic or si-FoxO3a-mediated facilitating effect (Figures 5(b) and 5(c), *P* < 0.05).

### 3.6. WSYQKL-Containing Serum Reversed miR-155 Mimic and si-FoxO3a Induced Migration on CSE-Mediated HASMCs

**Figure 6:**
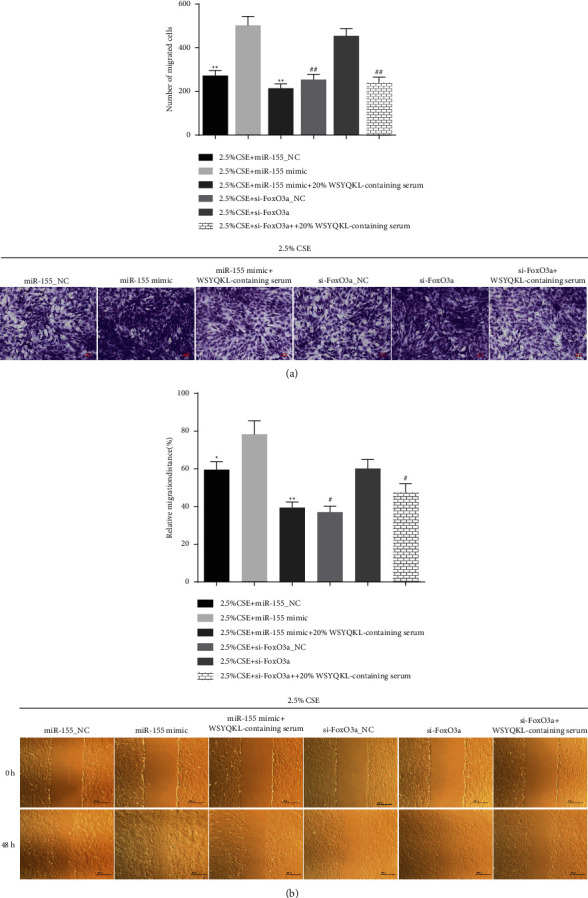
Effects of the WSYQKL-containing serum on cell migration in CSE-mediated hASMCs with miR-155 overexpression or FoxO3a knockdown. Cell migration was assessed after transfection with miR-155 mimic or si-FoxO3a in CSE-mediated hASMCs with 20% WSYQKL-containing serum treatment was assessed using the (a) Transwell and (b) wound healing assays. ^*∗*^*P* < 0.05, ^*∗∗*^*P* < 0.01*vs* 2.5% CSE + miR-155 mimic group. #*P* < 0.05, ##*P* < 0.01*vs* 2.5% CSE + si-FoxO3a group.

To observe the migration ability of hASMCs in each group, we performed a transwell assay and scarification assay. As illustrated in Figures 6(a) and 6(b), miR-155 mimic or FoxO3a silencing extremely enhanced cell migration in hASMCs induced by CSE (*P* < 0.05). Additionally, we discovered that WSYQKL-containing serum neutralized the facilitation of miR-155 mimic or FoxO3a silencing on CSE-triggered HASMC migration (Figure 6(a) and 6(b), *P* < 0.05).

### 3.7. WSYQKL-Containing Serum Reversed miR-155 Mimic and si-FoxO3a Induced Proliferation-Related Marker Levels in CSE-Triggered HASMCs

**Figure 7:**
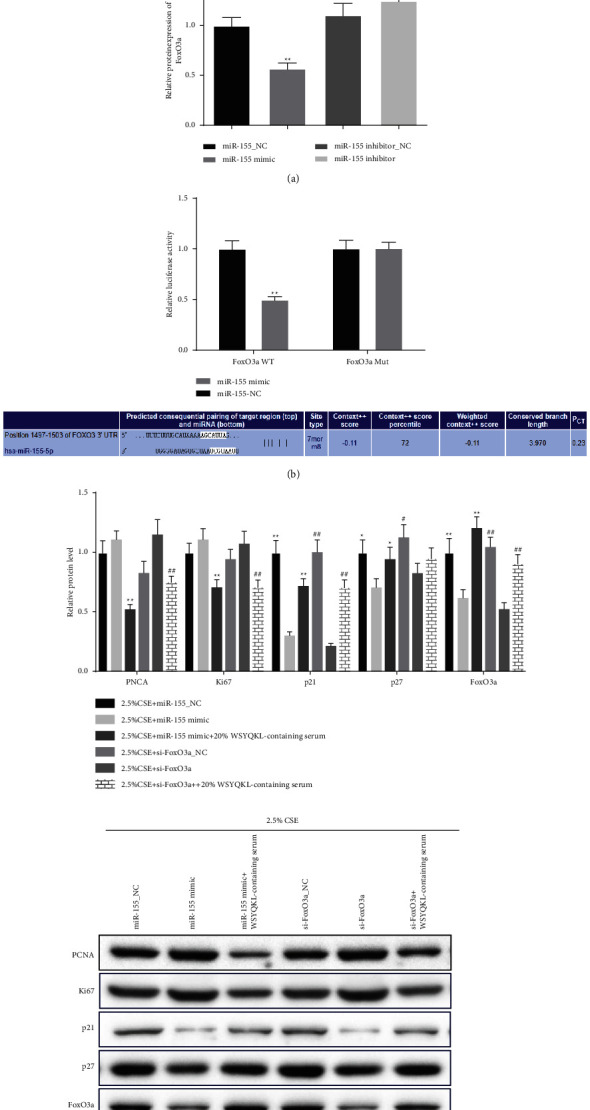
Effects of the WSYQKL-containing serum on cell proliferation-related protein levels in CSE-mediated HASMCs with miR-155 overexpression or FoxO3a knockdown. (a) Overexpression of miR-155 suppressed FoxO3a expression in HASMCs. (b) A predicted binding site of miR-155 within the FoxO3a 3′‐UTR region. Luciferase reporter assay was used to determine the direct interaction between miR-155 and FoxO3a 3′‐UTR. (c) Western blotting was used to detect the PCNA, Ki67, p21, p27, and FoxO3a protein expression levels in CSE-mediated hASMCs with 20% WSYQKL-containing serum treatment after transfection with miR-155 mimic or si-FoxO3a. ^*∗*^*P* < 0.05, ^*∗∗*^*P* < 0.01*vs* 2.5% CSE + miR-155 mimic group. #*P* < 0.05, ##*P* < 0.01*vs* 2.5% CSE + si-FoxO3a group.

In this part, miR-155 mimic, miR-155_NC, miR-155 inhibitor_NC, and miR-155 inhibitor were transfected into hASMCs. We discovered that miR-155 mimic effectively weakened FoxO3a level and miR-155 inhibitor slightly elevated FoxO3a expression in hASMCs (Figure 7(a), *P* < 0.01). In Figure 7(b), miR-155 mimics largely repressed the luciferase activity of FoxO3a WT but not that of FoxO3a Mut (*P* < 0.01). The results described by western blot that miR-155 mimic or FoxO3a silencing up-regulated the levels of PCNA and Ki67 and down-regulated the levels of p21, p27, and FoxO3a in CSE-triggered hASMCs, which was partially offset by WSYQKL-containing serum (Figure 7(c), *P* < 0.05).

## 4. Discussion

COPD can increase the risk of cardiovascular disease and has become the third leading reason of human death worldwide [18]. The occurrence of COPD is affected by external environmental factors and genetic factors; smoking is the commonest risk factor for COPD [19]. At present, the treatment schemes for COPD mostly take drugs, smoking cessation, and psychotherapy; oxygen inhalation and hormone therapy are required in severe cases, which affect the quality of life of patients [20,21]. Therefore, it is necessary to find an effective way to diagnose COPD.

The proliferation of ASMCs can cause tracheal wall thickening, airway remodeling, and hyperresponsiveness, which is one of the major pathogenesis of COPD [22]. Thus, probing the proliferation of ASMCs exhibits a significant effect on airway inflammation and airway remodeling of COPD. In the report of ASMCs cultured *in vitro*, it was uncovered that a certain concentration of CSE advanced the proliferation of ASMCs [23]. Our data were consistent with the previous study. Our study further pointed out that 2.5% of CSE obviously facilitated the migration of hASMCs. Importantly, this research first proved the modulation of WSYQKL on CSE-mediated hASMCs at the cellular level. The medicine serum of WSYQKL strongly alleviated the proliferation and migration of hASMCs induced by CSE, indicating that WSYQKL can alleviate the risk of COPD deterioration by weakening the proliferation and migration.

It has been reported that FoxO3a signaling is involved in the modulation of important activities such as cell proliferation, apoptosis, and migration [24,25]. FoxO3a activation can advance the expression of cell cycle inhibitory proteins p21 and p27, and affect cell proliferation and apoptosis [26,27]. By reviewing the literature, it was found that CSE induced the expressions of Ki67, PCNA, and p21 in bronchial epithelial cells and down-regulated the expression of FoxO3a in human lung adenocarcinoma cells [28,29]. Wylam et al. clarified that there was a high expression of PCNA in CSE-stimulated ASMCs [30]. Impaired FoxO3a activity has been described by some scholars as one of the intracellular responses activated by CSE [31]. Another study pointed out that FoxO3a was underexpressed in the lung tissue of COPD patients [32]. To further probe whether the antiproliferative and antimigratory effects of WSYQKL on CSE were due to the modulation of FoxO3a, we established FoxO3a-silenced hASMCs. Our findings found that WSYQKL-containing serum weakened the proliferation and migration of CSE-triggered hASMCs by repressing PCNA and Ki67 levels and advancing p21, p27, and FoxO3a levels, while FoxO3a silencing neutralized its effect.

By searching the literature on the relationship between FoxO3a and COPD, it was found that the Bufei Yishen formula protected COPD rats from PM2.5-mediated oxidative stress injury by modulating the miR-155/FoxO3a axis [33]. Moreover, Wu et al. reported that overexpression of miR-155 promoted the chronic intermittent hypoxia-induced NLRP3 inflammasome activation in renal tubular cells [34]. Additionally, Hawez et al. revealed that miR-155 regulates the pulmonary formation of neutrophil extracellular traps in abdominal sepsis [35]. More importantly, miR-155 has been noted to be highly expressed in COPD, while miR-155 deficiency notably suppressed CSE-mediated lung inflammation [36]. In the current study, we uncovered that CSE greatly enhanced the expression of miR-155 in hASMCs, whereas WSYQKL-containing serum evidently impeded the expression of miR-155 in hASMCs triggered by CSE. Multiple studies have demonstrated that FoxO3a is the target gene of miR-155 [37, 38]. Our study also confirmed the targeting relationship between miR-155 and FoxO3a. Furthermore, miR-155 mimic up-regulated the levels of PCNA and Ki67 and down-regulated the levels of p21, p27, and FoxO3a in CSE-triggered hASMCs, which was partially offset by WSYQKL-containing serum, indicating that the antiproliferative and antimigrate effects of WSYQKL on CSE-triggered hASMCs might be associated with an upregulation of FoxO3a by repressing miR-155.

Taken together, WSYQKL could suppress CSE-mediated proliferation and migration of hASMCs, and its mechanism might be related to the modulation of the miR-155/FOXO3a axis. Further study on the mechanism of WSYQKL modulating the miR-155/FOXO3a axis might become a novel method for the treatment of COPD.

## Data Availability

The datasets used and/or analyzed during the current study are available from the corresponding author upon reasonable request.

## Supplementary Materials

Supplementary Materials Supplementary Table S1. Chemical composition information of WSYQKL in ESI + mode. Supplementary Table S2. Chemical composition information of WSYQKL in ESI−mode.
