# Supplementary material for: Wenshen Yiqi Keli Mitigates the Proliferation and Migration of Cigarette Smoke Extract-Induced Human Airway Smooth Muscle Cells through miR-155/FoxO3a Axis
Source: Evid Based Complement Alternat Med. 2022 Nov 22;2022:4427637. doi: 10.1155/2022/4427637 (PMC9708348; doi:10.1155/2022/4427637)
Supplement: Supplementary Materials — Supplementary Table S1. Chemical composition information of WSYQKL in ESI + mode. Supplementary Table S2. Chemical composition information of WSYQKL in ESI−mode. [file 4427637.f1.zip › 4427637.f1/Table S1.doc]

**Table S1 Chemical composition information of WSYQKL in ESI+ mode**

| NO. | Component Name | Area | Retention Time | Formula | Precursor Mass | Found At Mass | Mass Error (ppm) | Library Score | Isotope Ratio Difference |
| --- | --- | --- | --- | --- | --- | --- | --- | --- | --- |
| 1 | L(+)-Arginine | 1034000 | 1.06 | C6H14N4O2 | 175.119 | 175.1188 | -1 | 95.7 | 3.5 |
| 2 | 1-Deoxynojirimycin | 251600 | 1.07 | C6H13NO4 | 164.092 | 164.0918 | 0.2 | 71.4 | 2.3 |
| 3 | Threonine | 94660 | 1.11 | C4H9NO3 | 120.066 | 120.0655 | 0 | 90.3 | 0.9 |
| 4 | Betaine | 255100 | 1.15 | C5H11NO2 | 118.086 | 118.0864 | 0.9 | 100 | 0.9 |
| 5 | Trigonelline | 426600 | 1.19 | C7H7NO2 | 138.055 | 138.0549 | -0.5 | 97.5 | 2.4 |
| 6 | Proline | 1413000 | 1.21 | C5H9NO2 | 116.071 | 116.0706 | -0.4 | 98 | 1.9 |
| 7 | Adenine | 82620 | 1.24 | C5H5N5 | 136.062 | 136.0619 | 0.8 | 99.2 | 0.2 |
| 8 | Nicotinic acid | 28490 | 1.76 | C6H5NO2 | 124.039 | 124.0394 | 1 | 93.4 | 1.5 |
| 9 | Adenosine | 259900 | 2.33 | C10H13N5O4 | 268.104 | 268.1039 | -0.4 | 98 | 5.5 |
| 10 | Guanosine | 22450 | 2.43 | C10H13N5O5 | 284.099 | 284.0992 | 0.9 | 100 | 3.8 |
| 11 | Phenylalanine | 52080 | 3.07 | C9H11NO2 | 166.086 | 166.0863 | 0.4 | 99.8 | 0.9 |
| 12 | Complanatoside | 3269 | 3.88 | C28H32O16 | 625.176 | 625.1767 | 0.7 | 100 | 8.9 |
| 13 | Ferulic Acid | 6183 | 4.03 | C10H10O4 | 195.065 | 195.0653 | 0.6 | 83.2 | 1.5 |
| 14 | Chlorogenic acid | 19590 | 4.33 | C16H18O9 | 355.102 | 355.1027 | 1 | 100 | 5.3 |
| 15 | Kaempferol-3-gentiobioside | 10320 | 4.51 | C27H30O16 | 611.161 | 611.1604 | -0.4 | 78.4 | 5.5 |
| 16 | Vitamin B2 | 6467 | 4.9 | C17H20N4O6 | 377.146 | 377.1453 | -0.7 | 83.1 | 3.2 |
| 17 | Daidzin | 1838 | 5.32 | C21H20O9 | 417.118 | 417.1176 | -1.1 | 96.2 | 12 |
| 18 | Calycosin-7-O-glucoside | 2395000 | 5.91 | C22H22O10 | 447.129 | 447.128 | -1.2 | 98.2 | 8.6 |
| 19 | Hyperin | 163000 | 5.98 | C21H20O12 | 465.103 | 465.1028 | 0 | 100 | 6.8 |
| 20 | β-Ecdysone | 988900 | 6.09 | C27H44O7 | 481.316 | 481.3156 | -0.8 | 74.4 | 10.7 |
| 21 | Sophoricoside | 4501 | 6.33 | C21H20O10 | 433.113 | 433.113 | 0.1 | 100 | 4.4 |
| 22 | Kaempferol | 197600 | 6.68 | C15H10O6 | 287.055 | 287.055 | -0.2 | 74.6 | 6.2 |
| 23 | Luteoloside | 164700 | 6.68 | C21H20O11 | 449.108 | 449.1079 | 0.2 | 100 | 6.9 |
| 24 | Rhoifolin | 15950 | 6.77 | C27H30O14 | 579.171 | 579.171 | 0.2 | 100 | 17.1 |
| 25 | Naringenin | 25880 | 6.86 | C15H12O5 | 273.076 | 273.0756 | -0.5 | 79.1 | 1.5 |
| 26 | Protopine | 124900 | 6.86 | C20H19NO5 | 354.134 | 354.1335 | -0.4 | 87.3 | 7.6 |
| 27 | Narirutin | 17170 | 6.86 | C27H32O14 | 581.186 | 581.1866 | 0.2 | 82.1 | 0.8 |
| 28 | Pratensein-7-O-glucoside | 49290 | 7.07 | C22H22O11 | 463.123 | 463.1236 | 0.2 | 85.7 | 7.5 |
| 29 | Hesperidin | 27770 | 7.1 | C28H34O15 | 611.197 | 611.1964 | -1.1 | 77.3 | 4.2 |
| 30 | Hesperetin | 17290 | 7.36 | C16H14O6 | 303.086 | 303.0863 | 0.1 | 84.4 | 2.9 |
| 31 | Corydaline | 49690 | 8.07 | C22H27NO4 | 370.201 | 370.2013 | -0.1 | 96.6 | 3.1 |
| 32 | Ononin | 2390000 | 8.24 | C22H22O9 | 431.134 | 431.1334 | -0.6 | 98.6 | 10.2 |
| 33 | Berberine | 43030 | 8.51 | C20H17NO4 | 336.123 | 336.123 | 0 | 97.8 | 8.5 |
| 34 | Tiliroside | 5692 | 8.76 | C30H26O13 | 595.145 | 595.1446 | -0.1 | 88.9 | 6.6 |
| 35 | 3-Hydroxy-9,10-Dimethoxypterocarpan | 869500 | 8.89 | C17H16O5 | 301.107 | 301.1068 | -0.8 | 87.3 | 7.7 |
| 36 | Calycosin | 887300 | 9.1 | C16H12O5 | 285.076 | 285.0755 | -0.8 | 95.5 | 6.6 |
| 37 | Cinnamic acid | 113200 | 10.51 | C9H8O2 | 149.06 | 149.0597 | -0.2 | 74.8 | 1.4 |
| 38 | Formononetin | 774200 | 12.17 | C16H12O4 | 269.081 | 269.0806 | -0.8 | 88.4 | 5.7 |
| 39 | Astragaloside Ⅳ | 57390 | 12.35 | C41H68O14 | 785.468 | 785.4676 | -0.7 | 80.7 | 12.1 |
| 40 | Nobiletin | 492200 | 13.88 | C21H22O8 | 403.139 | 403.1384 | -1 | 89.7 | 9.5 |
| 41 | Polyphyllin VI | 89580 | 15.15 | C39H62O13 | 739.426 | 739.4256 | -1 | 83.4 | 8.3 |
| 42 | Momordin Ic | 5439 | 17.22 | C41H64O13 | 782.469 | 782.4672 | -1.7 | 92.6 | 8.3 |
| 43 | Costunolide | 15980 | 17.9 | C15H20O2 | 233.154 | 233.1538 | 1 | 89.9 | 9.2 |
| 44 | Pogostone | 37680 | 18.14 | C12H16O4 | 225.112 | 225.1121 | 0 | 91.8 | 3.2 |
| 45 | Diosgenin | 63820 | 18.53 | C27H42O3 | 415.321 | 415.3208 | 0.2 | 95.8 | 8.4 |
| 46 | Diosgenin Glucoside | 33770 | 18.53 | C33H52O8 | 577.373 | 577.373 | -0.8 | 98.8 | 6 |
| 47 | Liriopesides B | 11680 | 18.53 | C39H62O12 | 723.431 | 723.4306 | -1.1 | 75 | 8 |
