# Supplementary material for: Wenshen Yiqi Keli Mitigates the Proliferation and Migration of Cigarette Smoke Extract-Induced Human Airway Smooth Muscle Cells through miR-155/FoxO3a Axis
Source: Evid Based Complement Alternat Med. 2022 Nov 22;2022:4427637. doi: 10.1155/2022/4427637 (PMC9708348; doi:10.1155/2022/4427637)
Supplement: Supplementary Materials — Supplementary Table S1. Chemical composition information of WSYQKL in ESI + mode. Supplementary Table S2. Chemical composition information of WSYQKL in ESI−mode. [file 4427637.f1.zip › 4427637.f1/Table S2.doc]

**Table S2 Chemical composition information of WSYQKL in ESI- mode**

| NO. | Component Name | Area | Retention Time | Formula | Precursor Mass | Found At Mass | Mass Error (ppm) | Library Score | Isotope Ratio Difference |
| --- | --- | --- | --- | --- | --- | --- | --- | --- | --- |
| 1 | L(+)-Arginine | 74260 | 1.05 | C6H14N4O2 | 173.104 | 173.1042 | -0.9 | 97.6 | 4.1 |
| 2 | Histidine | 17960 | 1.05 | C6H9N3O2 | 154.062 | 154.0622 | 0.1 | 80 | 3.1 |
| 3 | Glutamic acid | 16740 | 1.12 | C5H9NO4 | 146.046 | 146.0459 | -0.1 | 99.1 | 1.5 |
| 4 | L-Malic acid | 373700 | 1.27 | C4H6O5 | 133.014 | 133.0143 | 0.3 | 81 | 1 |
| 5 | Citric acid | 1906000 | 1.35 | C6H8O7 | 191.02 | 191.0197 | -0.3 | 98.7 | 2.1 |
| 6 | Amber Acid | 40330 | 2.31 | C4H6O4 | 117.019 | 117.0193 | -0.3 | 76 | 1 |
| 7 | Guanosine | 56860 | 2.43 | C10H13N5O5 | 282.084 | 282.0843 | -0.5 | 98 | 6.4 |
| 8 | Phenprobamate | 25700 | 3.08 | C9H11NO2 | 164.072 | 164.0717 | -0.2 | 95.2 | 1.6 |
| 9 | p-Coumaric acid | 86060 | 3.72 | C9H8O3 | 163.04 | 163.0399 | -1.3 | 88.1 | 3.1 |
| 10 | L-Tryptophan | 32500 | 3.94 | C11H12N2O2 | 203.083 | 203.0825 | -0.6 | 97.1 | 3.3 |
| 11 | Protocatechuic Aldehyde | 29880 | 4.39 | C7H6O3 | 137.024 | 137.0244 | -0.3 | 88.8 | 0.3 |
| 12 | Cryptochlorogenic acid | 105600 | 4.47 | C16H18O9 | 353.088 | 353.0875 | -1 | 96.6 | 7.3 |
| 13 | Eleutheroside E | 1046 | 5.35 | C34H46O18 | 787.267 | 787.2651 | -1.9 | 100 | 18.5 |
| 14 | Calycosin-7-o-glucoside | 2004000 | 5.91 | C22H22O10 | 491.119 | 491.1189 | -1.1 | 99.5 | 8.8 |
| 15 | Hyperin | 911200 | 5.98 | C21H20O12 | 463.088 | 463.0877 | -1.1 | 97.6 | 9.7 |
| 16 | β-Ecdysone | 2887000 | 6.08 | C27H44O7 | 525.307 | 525.3061 | -1.6 | 97.4 | 10.6 |
| 17 | Cistanoside D | 3503 | 6.4 | C31H40O15 | 697.235 | 697.234 | -1.3 | 76.1 | 1.9 |
| 18 | Luteoloside | 976300 | 6.68 | C21H20O11 | 447.093 | 447.0925 | -1.7 | 82.4 | 10.7 |
| 19 | Pinoresinol-glucoside | 54220 | 6.69 | C26H32O11 | 519.187 | 519.1862 | -1.9 | 96.8 | 4.5 |
| 20 | Naringin | 152600 | 6.86 | C27H32O14 | 579.172 | 579.171 | -1.6 | 96.4 | 13.1 |
| 21 | 3,5-DCQA Isochlorogenic acid A | 9597 | 7.09 | C25H24O12 | 515.119 | 515.1182 | -2.4 | 97.7 | 6 |
| 22 | Phloridzin | 3706 | 7.25 | C21H24O10 | 435.13 | 435.1287 | -2.1 | 82.6 | 2 |
| 23 | Hesperidin | 131500 | 7.36 | C28H34O15 | 609.182 | 609.1817 | -1.3 | 99.1 | 13.7 |
| 24 | Salvianolic acid B | 26870 | 7.85 | C36H30O16 | 717.146 | 717.1448 | -1.8 | 88 | 11.8 |
| 25 | Baicalin | 11780 | 8.16 | C21H18O11 | 445.078 | 445.0768 | -1.9 | 100 | 0.9 |
| 26 | Ononin | 1434000 | 8.24 | C22H22O9 | 475.125 | 475.124 | -1.1 | 99.3 | 10.4 |
| 27 | Tiliroside | 12860 | 8.77 | C30H26O13 | 593.13 | 593.1289 | -1.9 | 72.8 | 4 |
| 28 | Quercetin | 6041 | 9.03 | C15H10O7 | 301.035 | 301.0347 | -2.1 | 70.9 | 7.2 |
| 29 | Calycosin | 562600 | 9.11 | C16H12O5 | 283.061 | 283.0605 | -2.6 | 80.1 | 7.6 |
| 30 | Isomucronulatol-7-O-glucoside | 516700 | 9.2 | C23H28O10 | 463.161 | 463.1598 | -2.5 | 95.2 | 9.9 |
| 31 | 3-Hydroxy-9,10-Dimethoxypterocarpan | 14380 | 9.78 | C17H16O5 | 299.092 | 299.0918 | -2.2 | 77.9 | 4.3 |
| 32 | Formononetin | 485400 | 12.17 | C16H12O4 | 267.066 | 267.0658 | -1.9 | 91.6 | 9.4 |
| 33 | Astragaloside Ⅳ | 462100 | 12.34 | C41H68O14 | 829.459 | 829.4569 | -2.7 | 88.5 | 12.7 |
| 34 | Chikusetsusponin IVa | 2247 | 12.69 | C42H66O14 | 793.438 | 793.4359 | -2.7 | 89.9 | 7.9 |
| 35 | Astragaloside Ⅱ | 716200 | 13.5 | C43H70O15 | 871.47 | 871.468 | -1.9 | 71.9 | 19.8 |
| 36 | Saikosaponin A | 2513 | 13.99 | C42H68O13 | 825.464 | 825.4623 | -2.3 | 99.7 | 7.5 |
| 37 | Liriope muscari baily saponins C | 3596000 | 15.14 | C44H70O17 | 915.46 | 915.4586 | -0.9 | 76.7 | 18.4 |
| 38 | Momordin Ic | 57570 | 17.21 | C41H64O13 | 763.427 | 763.426 | -1.8 | 100 | 9.4 |
| 39 | Gracillin | 341700 | 18.51 | C45H72O17 | 929.475 | 929.474 | -1.2 | 96.8 | 16.8 |
| 40 | PolyphyllinⅠ | 101600 | 18.7 | C44H70O16 | 899.465 | 899.4639 | -0.8 | 97.2 | 16.2 |
| 41 | Liriopesides B | 8422 | 19.17 | C39H62O12 | 767.422 | 767.4217 | -0.9 | 92.6 | 2.1 |
| 42 | Gingerglycolipid B | 8981 | 19.23 | C33H58O14 | 723.381 | 723.3803 | -0.8 | 96.1 | 11.7 |
